# Supplementary material for: Impaired bone morphogenetic protein (BMP) signaling pathways disrupt decidualization in endometriosis
Source: Commun Biol. 2024 Feb 24;7:227. doi: 10.1038/s42003-024-05898-z (PMC10894266; doi:10.1038/s42003-024-05898-z)
Supplement: Supplementary file 1 — Supplementary Information [file 42003_2024_5898_MOESM1_ESM.pdf]

SUPPLEMENTARY INFORMATION

Impaired bone morphogenetic protein (BMP) signaling pathways disrupt decidualization in endometriosis

Zian Liao[1,2,3,4,#], Suni Tang[1,4,#], Peixin Jiang[1,5], Ting Geng[1], Dominique I. Cope[1,4], Timothy N. Dunn[6,7], Joie Guner[8], Linda Alpuing Radilla[6], Xiaoming Guan[6], Diana Monsivais[1,4,*]

[1]Department of Pathology & Immunology, Baylor College of Medicine, Houston, TX, 77030, USA

[2]Department of Molecular and Human Genetics, Baylor College of Medicine, Houston, TX, 77030, USA

[3]Graduate Program of Genetics and Genomics, Baylor College of Medicine, Houston, TX, 77030, USA

[4]Center for Drug Discovery, Baylor College of Medicine, Houston, TX, 77030, USA

[5]Department of Thoracic/Head and Neck Medical Oncology, the University of Texas MD Anderson Cancer Center, Houston, TX, 77030, USA.

[6]Department of Obstetrics and Gynecology, Baylor College of Medicine, Houston, TX, 77030, USA

[7]Division of Reproductive Endocrinology & Infertility, Baylor College of Medicine, Houston, TX, 77030, USA

[8]Department of Obstetrics and Gynecology, Division of Reproductive Endocrinology and Infertility, University of Southern California, Los Angeles, CA, 90033, USA

[#] These authors contributed equally

**Correspondence addressed to:*

Diana Monsivais
Baylor College of Medicine
One Baylor Plaza, Smith S217
Houston, TX 77030
dmonsiva@bcm.edu
ORCID ID: 0000-0001-5660-6392

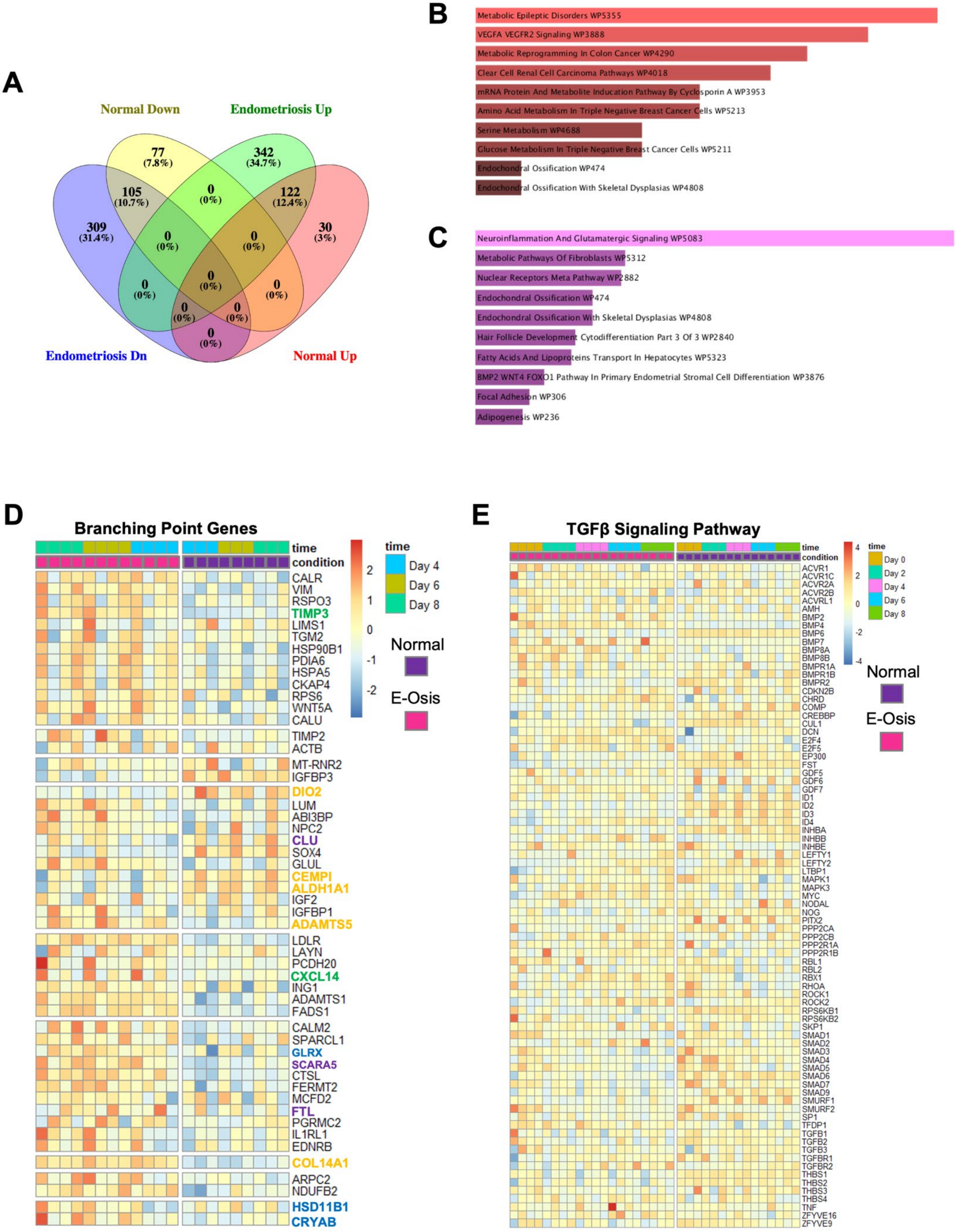
A
Normal Down
Endometriosis Up
Endometriosis Dn
Normal Up
77 (7.8%)
342 (34.7%)
105 (10.7%)
0 (0%)
122 (12.4%)
309 (31.4%)
30 (3%)
B
Metabolic Epileptic Disorders WP5355
VEGFA VEGFR2 Signaling WP3888
Metabolic Reprogramming In Colon Cancer WP4290
Clear Cell Renal Cell Carcinoma Pathways WP4018
mRNA Protein And Metabolite Inducation Pathway By Cyclosporin A WP3953
Amino Acid Metabolism In Triple Negative Breast Cancer Cells WP5213
Serine Metabolism WP4688
Glucose Metabolism In Triple Negative Breast Cancer Cells WP5211
Endochondral Ossification WP474
Endochondral Ossification With Skeletal Dysplasias WP4808
C
Neuroinflammation And Glutamatergic Signaling WP5083
Metabolic Pathways Of Fibroblasts WP5312
Nuclear Receptors Meta Pathway WP2882
Endochondral Ossification WP474
Endochondral Ossification With Skeletal Dysplasias WP4808
Hair Follicle Development Cytodifferentiation Part 3 Of 3 WP2840
Fatty Acids And Lipoproteins Transport In Hepatocytes WP5323
BMP2 WNT4 FOXO1 Pathway In Primary Endometrial Stromal Cell Differentiation WP3876
Focal Adhesion WP306
Adipogenesis WP236
D
Branching Point Genes
time
condition
time
Day 4
Day 6
Day 8
Normal
E-Osis
2
1
0
-1
-2
CALR
VIM
RSPO3
TIMP3
LIMS1
TGM2
HSP90B1
PDIA6
HSPA5
CKAP4
RPS6
WNT5A
CALU
TIMP2
ACTB
MT-RNR2
IGFBP3
DIO2
LUM
ABI3BP
NPC2
CLU
SOX4
GLUL
CEMPI
ALDH1A1
IGF2
IGFBP1
ADAMTS5
LDLR
LAYN
PCDH20
CXCL14
ING1
ADAMTS1
FADS1
CALM2
SPARCL1
GLRX
SCARA5
CTSL
FERMT2
MCFD2
FTL
PGRMC2
IL1RL1
EDNRB
COL14A1
ARPC2
NDUFB2
HSD11B1
CRYAB
E
TGFβ Signaling Pathway
time
condition
4
2
0
-2
-4
time
Day 0
Day 2
Day 4
Day 6
Day 8
Normal
E-Osis
ACVR1
ACVR1C
ACVR2A
ACVR2B
ACVRL1
AMH
BMP2
BMP4
BMP6
BMP7
BMP8A
BMP8B
BMPR1A
BMPR1B
BMPR2
CDKN2B
CHRD
COMP
CREBBP
CUL1
DCN
E2F4
E2F5
EP300
FST
GDF5
GDF6
GDF7
ID1
ID2
ID3
ID4
INHBA
INHBB
INHBE
LEFTY1
LEFTY2
LTBP1
MAPK1
MAPK3
MYC
NODAL
NOG
PITX2
PPP2CA
PPP2CB
PPP2R1A
PPP2R1B
RBL1
RBL2
RBX1
RHOA
ROCK1
ROCK2
RPS6KB1
RPS6KB2
SKP1
SMAD1
SMAD2
SMAD3
SMAD4
SMAD5
SMAD6
SMAD7
SMAD9
SMURF1
SMURF2
SP1
TFDP1
TGFB1
TGFB2
TGFB3
TGFBR1
TGFBR2
THBS1
THBS2
THBS3
THBS4
TNF
ZFYVE16
ZFYVE9

Supplemental Figure 1. Transcriptomic, gene ontology analysis and correlation with decidualization branchpoints of the time course decidualization datasets. A) A Venn diagram was used to display the number of conserved genes between the normal and endometriosis groups during the time course decidualization program. Genes that showed a significant change on Day 8 of decidualization were used (>1.4, <0.4-fold change, FDR < 0.05). B-C) Gene ontology analysis of the genes that showed a significant change (>1.4, <0.4-fold change, FDR < 0.05) on Day 8 of decidualization in the normal (B) or endometriosis (C) groups. D) DEGs from the Day 4, 6, 8 EPC treatment in normal and endometriosis stromal cells were correlated with the top 50 genes indicative of the senescence and non-senescent decidual branchpoints from Lucas ES et al. E) Heatmap displaying the gene expression levels of TGFβ family members in stromal cells from normal and endometriosis donors induced to decidualize over time.

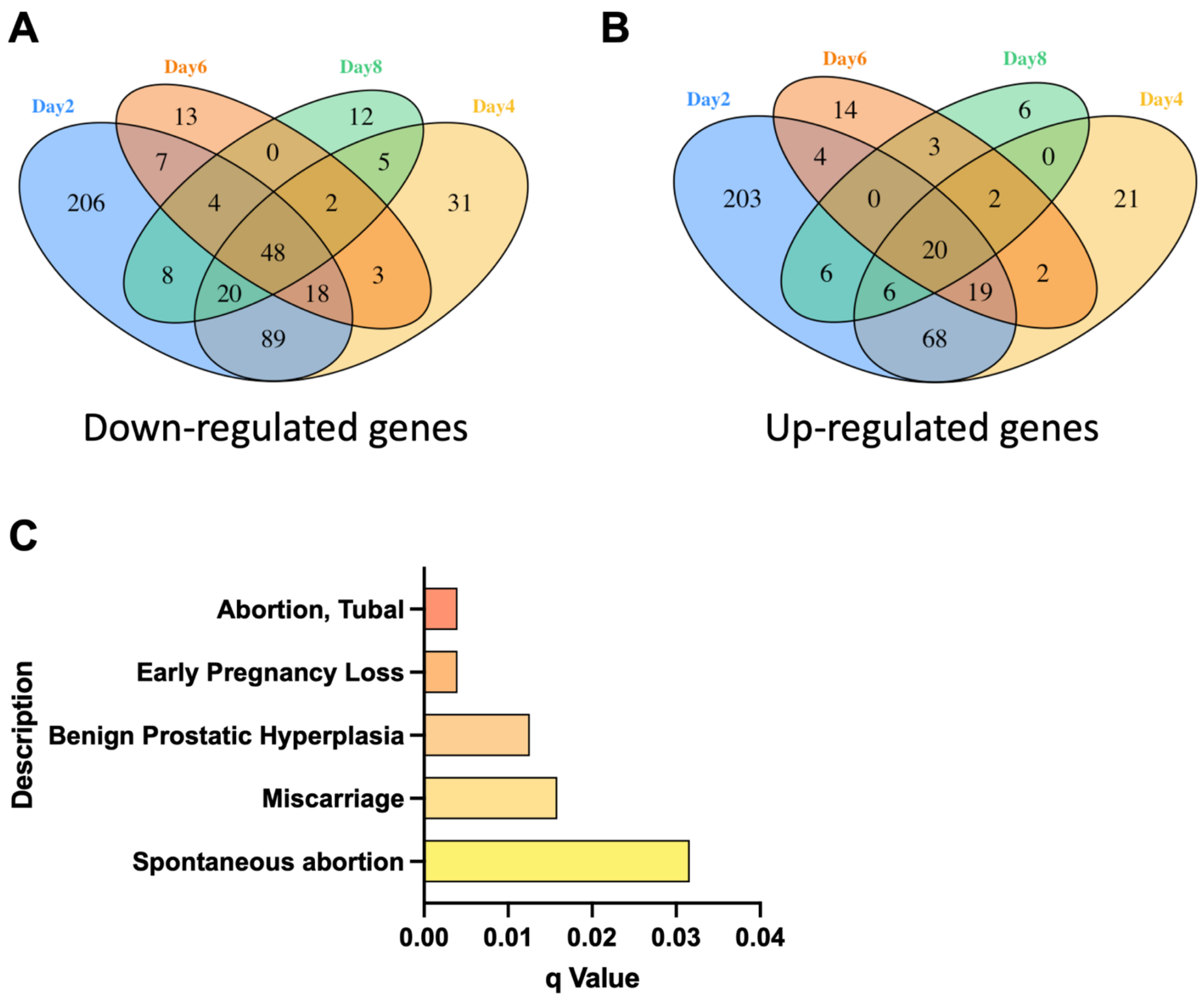

Supplemental Figure 2. Transcriptomic and classification of genes involved in the decidualization of endometrial stromal cells from individuals with and without endometriosis. A-B) A Venn diagram was used to display the number of conserved downregulated (A) and upregulated (B) genes in the time course decidualization of endometrial stromal cells from individuals with endometriosis. The analysis identified that 48 genes were consistently down-regulated (A) and 20 genes were consistently up-regulated (B) regardless of the EPC treatment length. C) DisGeNET analysis for the 48 genes that were consistently down regulated in the stromal cells from individuals with endometriosis relative to individuals without endometriosis during the time course decidualization treatment.

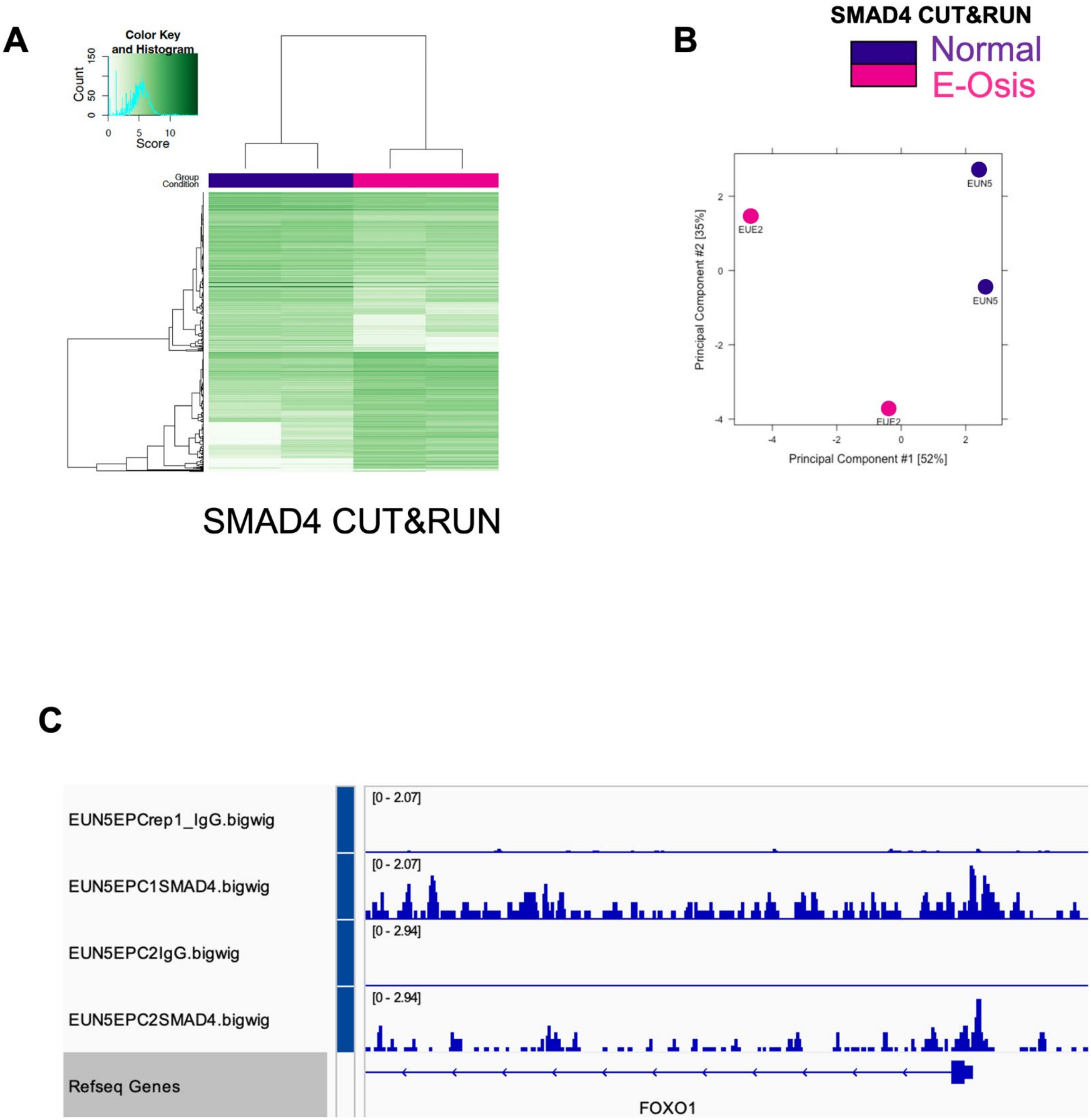

Supplemental Figure 3. Patient-specific distribution of SMAD4 binding analysis during decidualization. A) SMAD4 CUT&RUN in endometrial stromal cells from individuals with and without endometriosis (E-Osis) after 4 days of EPC treatment. Differential peak signals obtained for the genome-wide SMAD4 distribution in normal versus endometriosis. B) PCA plot of the SMAD4 binding signals comparing the normal versus endometriosis sample replicates. C) Genome track view of the SMAD4 peaks located to the *FOXO1* TSS and gene body in stromal cells from normal individuals induced to decidualize for 4 days with EPC.

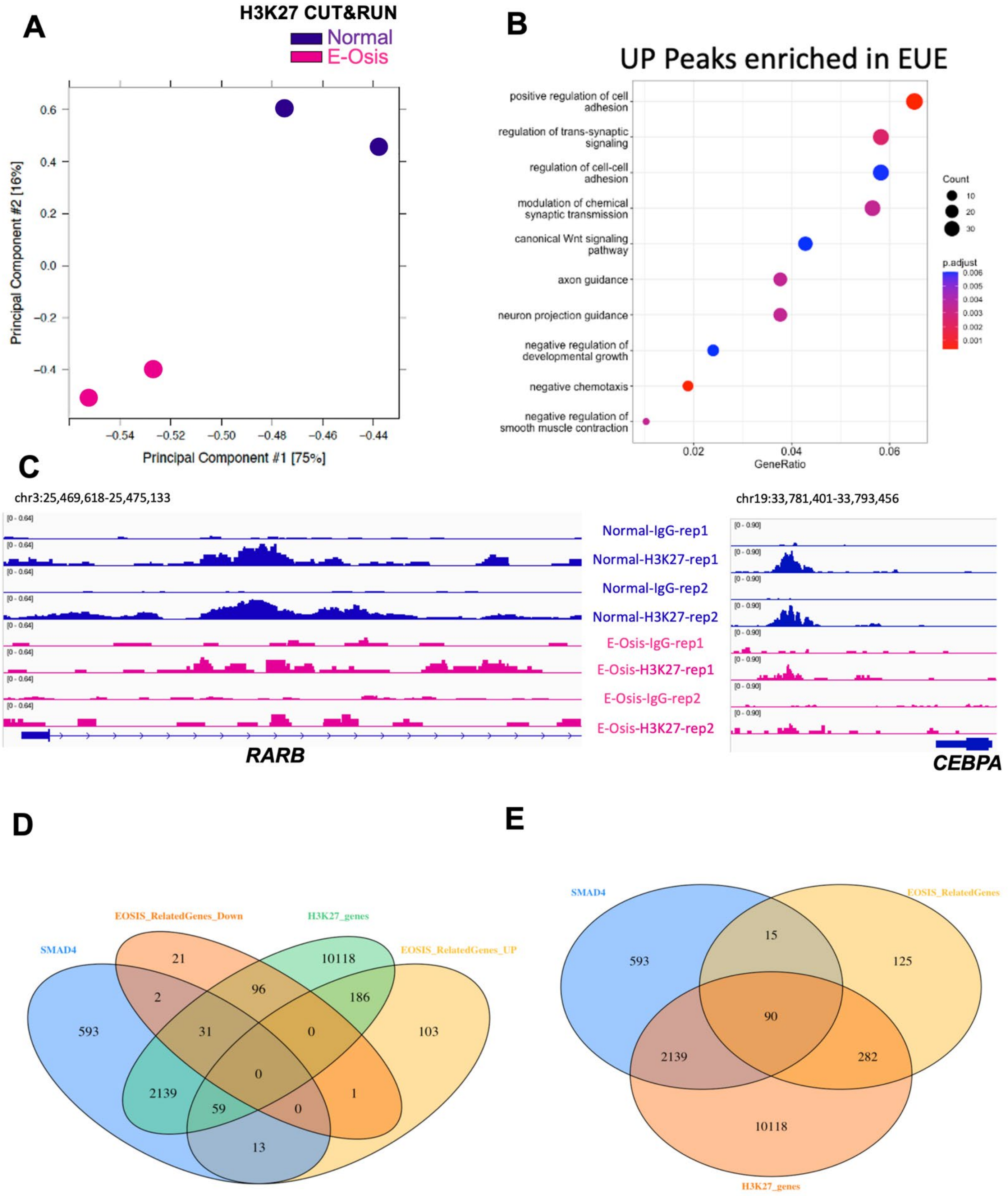

Supplemental Figure 4. Analysis of H3K27ac in the decidualizing stromal cells of individuals with and without endometriosis. A) PCA plot for H3K27ac replicates of normal and endometriosis stromal cells, showing that the replicates are reproducible. B) Gene ontology classification of the H3K27ac peaks increased

in endometriosis stromal cells, showing that categories related to the regulation of cell adhesion were overrepresented. C) Genome track views for the distribution of H3K27ac peaks in the promoter regions of *RARB* and *CEBPA,* showing increased peak density in the endometrial stromal cells from individuals without endometriosis. D-E) Comparison of endometriosis-associated DEGs with SMAD4 and H3K27 bound genes. The endometriosis dataset was used from Day 4 EPC (FC>1.4, <0.4, FDR<0.05). The genes are visualized as Venn diagrams and grouped by Up- and Down-regulated (D) or as a Venn diagram without distinguishing between Up or Down-regulated genes (E).

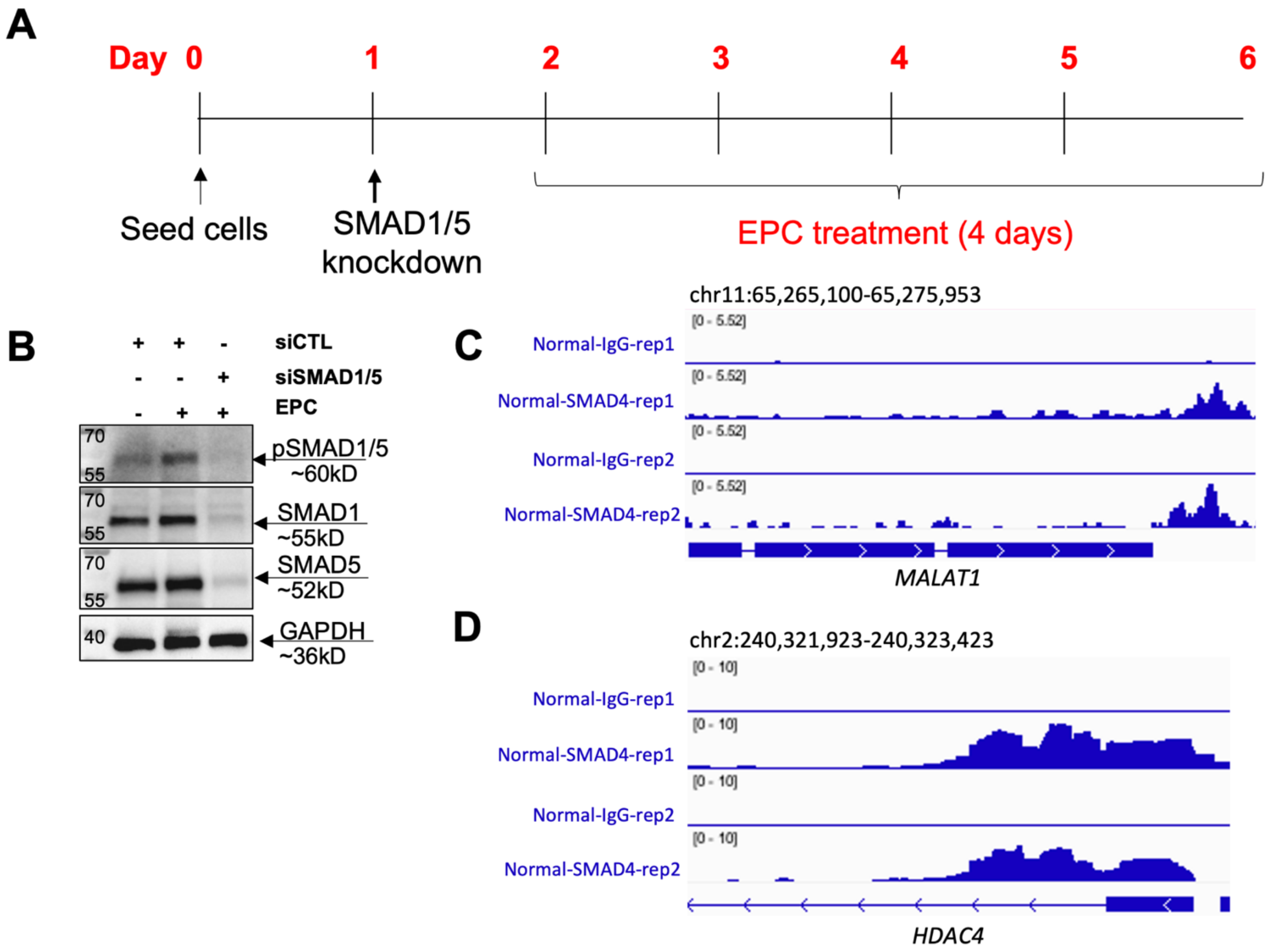

Supplemental Figure 5. siRNA knockdown of SMAD1 and SMAD5 perturbs decidualization. A) Experimental layout showing the time points at which endometrial stromal cells were plated, transfected with SMAD1/SMAD5 siRNAs, and induced to decidualize with 35nM estradiol, 1µM medroxyprogesterone acetate and 50µM cAMP (EPC) for 4 days. B) Western blot of endometrial stromal cells treated with non-targeting siRNAs (siCTL) or siRNAs targeting SMAD1 and SMAD5 and EPC. Membranes were probed with pSMAD1/5, total SMAD1, total SMAD5 and GAPDH antibodies to confirm knockdown of SMAD1/5 was successful. C-D) Genome track views of the *MALAT1* and *HDAC4* genes showing enrichment of the SMAD4 peaks.

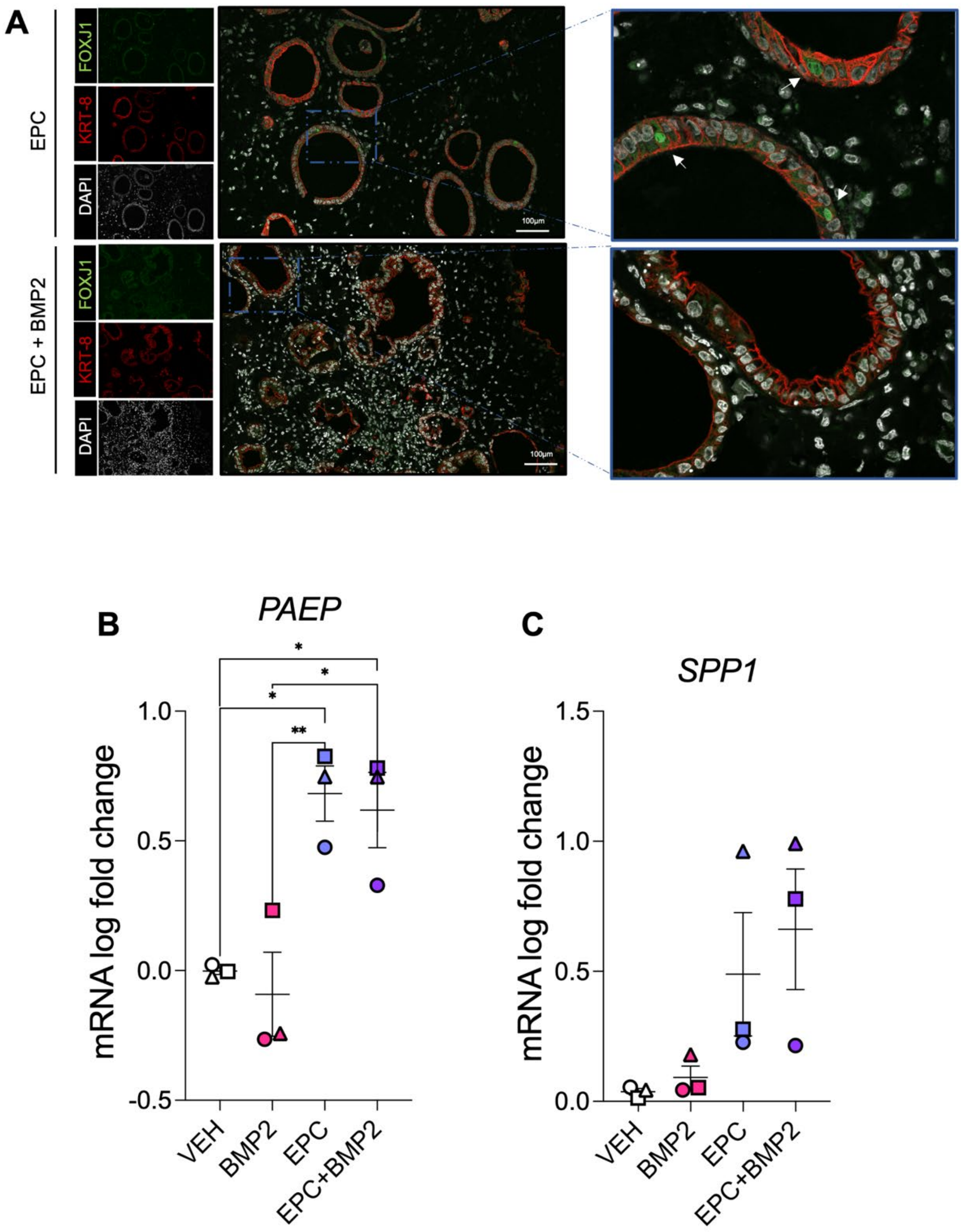

Supplemental Figure 6. Analysis of the ciliated cell marker, FOXJ1, in decidualizing 3D endometrial assembloids from donors with endometriosis. A) Immunostaining of the ciliated cell marker, FOXJ1 (green), the epithelial cell marker (cytokeratin 8, KRT-8, red) and DAPI (white) in endometrial assembloids treated with EPC or EPC + BMP2, the hormonal stimuli. B-C) qRT-PCR analysis of glandular decidualization markers, *PAEP* (B) or *SPP1* (D) in the endometrial assembloids treated with Vehicle, BMP2, EPC, or EPC + BMP2. Plotted values represent mean +/- standard error of the mean, with the different symbols corresponding to each patient's trajectory. Data were analyzed using a one-way ANOVA with a Tukey's posthoc test.

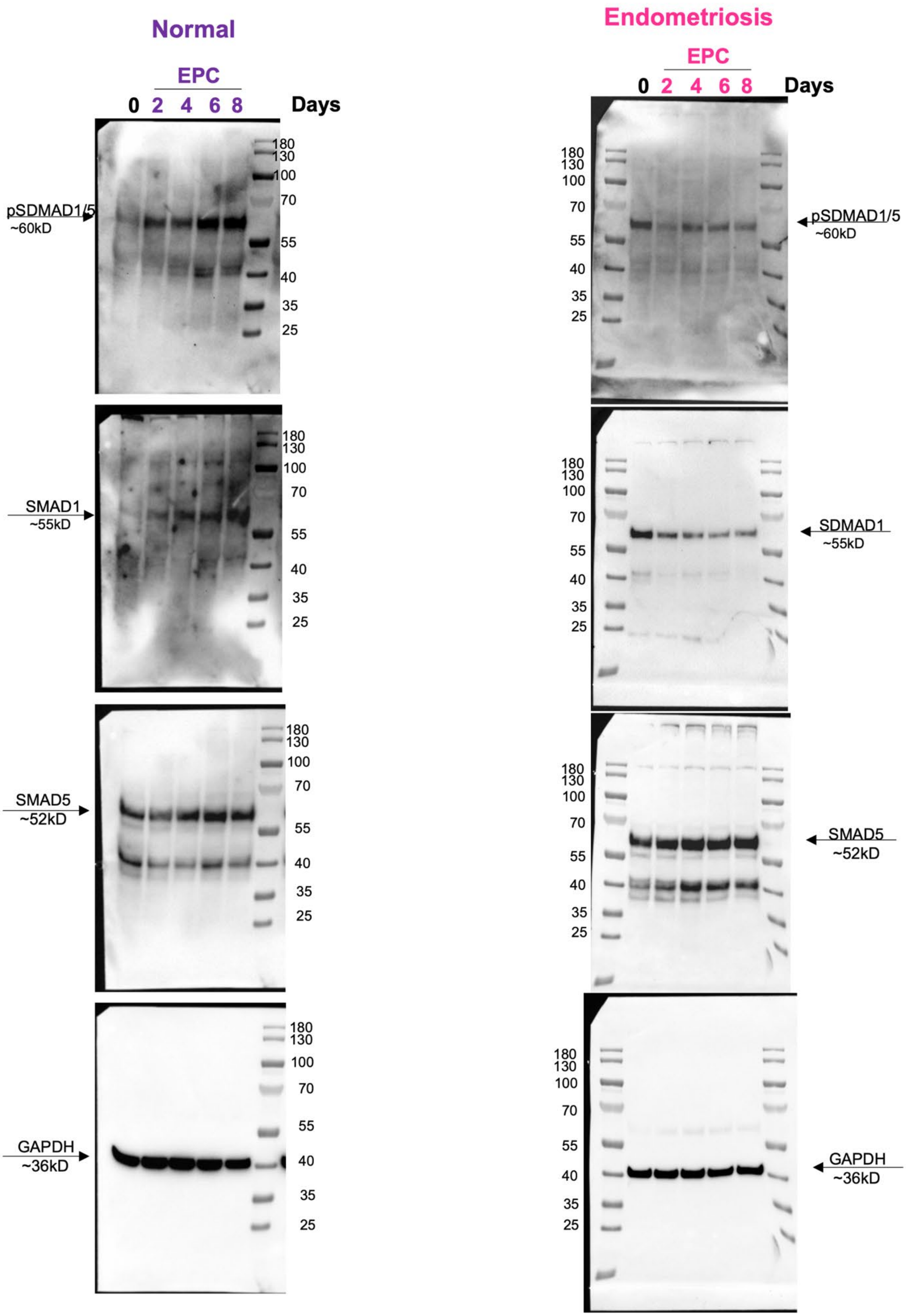

Supplemental Figure 7. Uncropped western blot images corresponding to Figure 3.

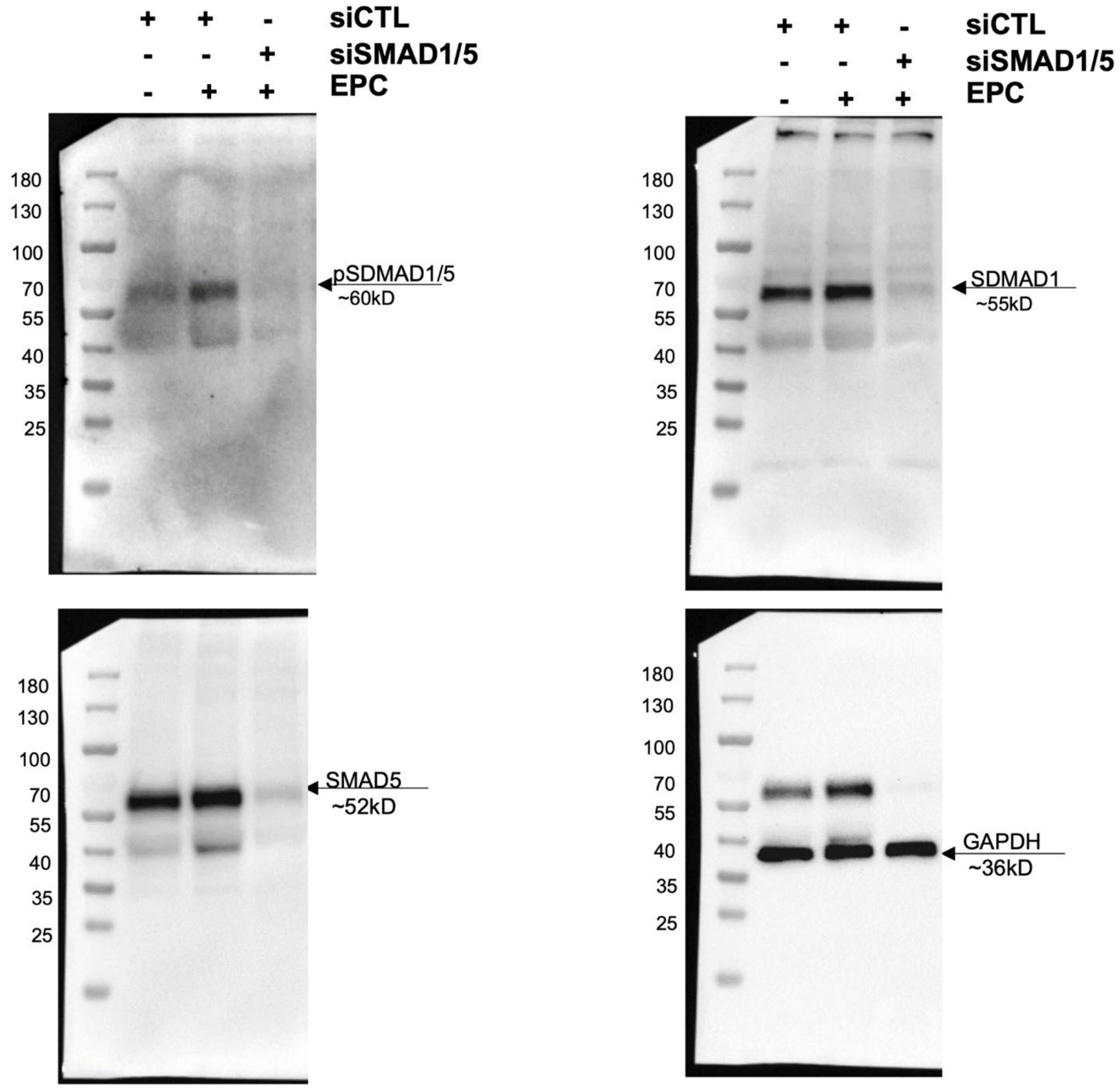

Supplemental Figure 8. Uncropped western blot images corresponding to Supplemental Figure 5.

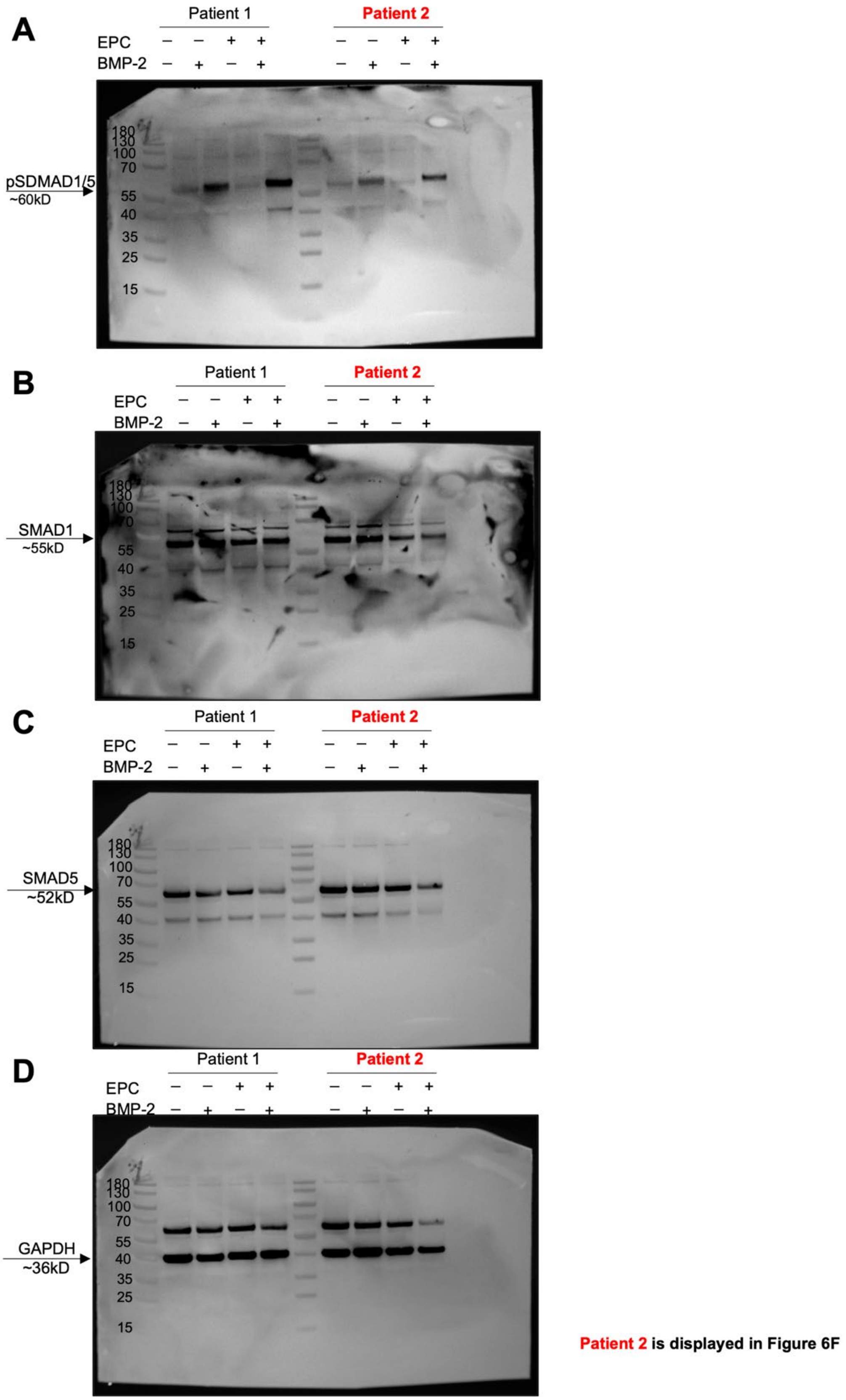

Supplemental Figure 9. Uncropped western blot images corresponding to Figure 6.

SUPPLEMENTAL TABLES and DATA FILES

Supplemental Table 1. qRT-PCR Primer Sequences

Gene	Forward (5'-3')	Reverse (5'-3')
BMP2	ACCCGCTGTCTCTAGCGT	TTTCAGGCCGAACATGCTGAG
IGFBP1	TTGGGACGCCATCAGTACCTA	TTGGCTAAACTCTCTACGACTCT
PRL	AAGCTGTAGAGATTGAGGAGCAAAC	TCAGGATGAACCTGGCTGACTA
FOXO1	TGATAACTGGAGTACATTTCGCC	CGGTCATAATGGGTGAGAGTCT
WNT4	CTCCACACTCGACTCCTTGC	CCGAAGAGATGGCGTACACG
SPP1	TGCAGCCTTCTCAGCCAAA	GGAGGCAAAAGCAAATCACTG
GAPDH	ACAACTTTGGTATCGTGGAAGG	GCCATCACGCCACAGTTTC

Supplemental Table 2. Antibody list

Antibody	Vendor	Catalog	Application
pSMAD1/5	Cell Signaling	9516	WB 1:1000
SMAD1	Invitrogen	38-5400	WB 1:1000
SMAD5	Proteintech	12167-1-AP	WB 1:1000
GAPDH	Proteintech	HRP-60004	WB 1:5000
Vimentin	Cell Signaling	5741	IF 1:200
SMAD4	Abcam	ab40759	CUT&RUN 1:50
K3K27ac	Cell Signaling	8173	CUT & RUN 1:50
KRT-8	DSHB	TROMA-I	IF 1:50
FOXJ1	Sigma	HPA005714	IF 1:100
Antibody-Rabbit IgG (H+L) Highly Cross-Adsorbed_Alexa Fluor 488	ThermoFisher	A-21206	IF 1:250
Antibody-Rat IgG (H+L) Highly Cross-Adsorbed_Alexa Fluor 594	ThermoFisher	A-21209	IF 1:250
