# Supplementary material for: Impaired bone morphogenetic protein (BMP) signaling pathways disrupt decidualization in endometriosis
Source: Commun Biol. 2024 Feb 24;7:227. doi: 10.1038/s42003-024-05898-z (PMC10894266; doi:10.1038/s42003-024-05898-z)
Supplement: Supplementary file 2 — Description of Additional Supplementary Files [file 42003_2024_5898_MOESM2_ESM.pdf]

Description of Additional Supplementary Files

File name: Supplementary Data 1
Description: Differentially expressed genes in endometrial stromal cells from individuals with and without endometriosis during the time course decidualization treatment compared to baseline Day 0.

File name: Supplementary Data 2
Description: Gene ontology classification of differentially expressed genes in the endometrial stromal cells from individuals with and without endometriosis during time course decidualization compared to baseline Day 0.

File name: Supplementary Data 3
Description: Transcription factor regulators determined by EnrichR analysis from the genes that were differentially expressed during decidualization in endometrial stromal cells from individuals with and without endometriosis relative to baseline Day 0.

File name: Supplementary Data 4
Description: Complete gene expression matrix comparing endometrial stromal cells from individuals with and without endometriosis at each time point during decidualization and the DisGeNET analysis on the 48 consistently down regulated genes.

File name: Supplementary Data 5
Description: SMAD4 peak annotations in endometrial stromal cells from individuals with and without endometriosis treated with EPC for 4 days.

File name: Supplementary Data 6
Description: Peak annotation and KEGG GO analysis of H3K27ac in endometrial stromal cells from with and without endometriosis treated with EPC for 4 days. Correlation of SMAD4 and H3K27Ac peaks with the DEGs from endometriosis vs. normal EPC treated cells for 4 days.

File name: Supplementary Data 7
Description: Genes that are differentially regulated following EPC treatment and SMAD1/5 siRNA knockdown (siCTL + EPC vs. siSMAD1/5 + EPC).

File name: Supplementary Data 8
Description: The source data behind the graphs in the paper.
