# Supplementary material for: Impaired bone morphogenetic protein (BMP) signaling pathways disrupt decidualization in endometriosis
Source: Commun Biol. 2024 Feb 24;7:227. doi: 10.1038/s42003-024-05898-z (PMC10894266; doi:10.1038/s42003-024-05898-z)
Supplement: Supplementary file 11 — Reporting Summary [file 42003_2024_5898_MOESM11_ESM.pdf]

nature portfolio

Corresponding author(s): Diana Monsivais

Last updated by author(s): Jan 22, 2024

Reporting Summary

Nature Portfolio wishes to improve the reproducibility of the work that we publish. This form provides structure for consistency and transparency in reporting. For further information on Nature Portfolio policies, see our Editorial Policies and the Editorial Policy Checklist.

Statistics

For all statistical analyses, confirm that the following items are present in the figure legend, table legend, main text, or Methods section.

n/a	Confirmed	
☐	☒	The exact sample size (n) for each experimental group/condition, given as a discrete number and unit of measurement
☐	☒	A statement on whether measurements were taken from distinct samples or whether the same sample was measured repeatedly
☐	☒	The statistical test(s) used AND whether they are one- or two-sided<br>*Only common tests should be described solely by name; describe more complex techniques in the Methods section.*
☐	☒	A description of all covariates tested
☐	☒	A description of any assumptions or corrections, such as tests of normality and adjustment for multiple comparisons
☐	☒	A full description of the statistical parameters including central tendency (e.g. means) or other basic estimates (e.g. regression coefficient) AND variation (e.g. standard deviation) or associated estimates of uncertainty (e.g. confidence intervals)
☐	☒	For null hypothesis testing, the test statistic (e.g. F, t, r) with confidence intervals, effect sizes, degrees of freedom and P value noted<br>*Give P values as exact values whenever suitable.*
☒	☐	For Bayesian analysis, information on the choice of priors and Markov chain Monte Carlo settings
☒	☐	For hierarchical and complex designs, identification of the appropriate level for tests and full reporting of outcomes
☒	☐	Estimates of effect sizes (e.g. Cohen's d, Pearson's r), indicating how they were calculated

Our web collection on statistics for biologists contains articles on many of the points above.

Software and code

Policy information about availability of computer code

Data collection	RNA Sequencing data are available in the Gene Expression Omnibus (GSE243158).<br>CUT and RUN: Sequencing data are available in the Gene Expression Omnibus (GSE243158).
Data analysis	RNAseq: RNA expression profiles were obtained at each timepoint using RNA sequencing analyses (20-30 million paired-end reads using NovaSeq System from Novogene Corporation Inc. Reads were trimmed with fastp v0.23.2 and aligned using STAR 2.7.10a to human genome assembly GRCh38.p13. Differentially expressed genes between normal patients and endometriosis EPC-treated cells were obtained by comparing to the baseline samples (Day 0). Significantly changed genes during the time course treatment were obtained using an ANOVA F-test using an FDR <0.05 from patients without (n=3) and with endometriosis (n=4). All DEGs are presented in Supplementary Table 1. EnrichR1 was used to identify the gene ontology classifications (Supplementary Table 2), as well as ENCODE and ChEA consensus transcription factors known to regulate differentially expressed genes in the normal and endometriosis EPC-treated stromal cells (Supplementary Table 3). Differentially expressed genes between normal and endometriosis stromal cells were obtained by comparing transcripts at each time point of treatment. Differentially expressed genes between normal (n=4) and endometriosis (n=3) were identified using a Wald test with a cutoff values of fold-change > 2 or < 1/2 and FDR < 0.05 (Supplementary Table 4). Gene and pathway enrichment analysis was conducted using R package Cluster Profiler.<br>CUT&RUN: Sequencing libraries were prepared using NEBNext Ultra II DNA Library Prep Kit (New England BioLabs, Cat #E7645) following manufacture's protocol. Paired-end 150bp sequencing was performed on a NEXTSeq550 (Illumina) platform and each sample was targeted for 10 million reads. Sequencing raw data were de-multiplexed by bcl2fastq v2.20 with fastqc for quality control and then mapped to reference genome hg19 by Bowtie2, with parameters of --end-to-end --very-sensitive --no-mixed --no-discordant --phred33 -I 10 -X 700. For Spike-in mapping, reads were mapped to E. coli genome U00096.3. Spike-in normalization was achieved through multiply primary genome coverage by scale factor (100000 / fragments mapped to E. coli genome). CUT&RUN peaks were called by Model-based Analysis of ChIP-Seq

(MACS/2.0.10) with the parameters of -f BAMPE -g 1.87e9 -q 0.05 (H3K27ac) or -q 0.1 (SMAD4). Track visualization was done by bedGraphToBigWig20, bigwig files were imported to Integrative Genomics Viewer for visualization. For peak annotation, genomic coordinates were annotated by ChIPseeker. Differential binding analysis and clustering were conducted using DiffBind. Direct targets motif analysis was conducted through Binding and Expression Target Analysis (BETA)92 with parameter BETA plus –p –e –k LIM –g hg19 --gs hg19.fa --bl. Gene and pathway enrichment analysis was conducted using R package Cluster Profiler110. Annotated peak files were included in Supplemental Table 5 (SMAD4) and Supplemental Table 6 (H3K27ac).

For manuscripts utilizing custom algorithms or software that are central to the research but not yet described in published literature, software must be made available to editors and reviewers. We strongly encourage code deposition in a community repository (e.g. GitHub). See the Nature Portfolio guidelines for submitting code & software for further information.

Data

Policy information about availability of data

All manuscripts must include a data availability statement. This statement should provide the following information, where applicable:

- Accession codes, unique identifiers, or web links for publicly available datasets
- A description of any restrictions on data availability
- For clinical datasets or third party data, please ensure that the statement adheres to our policy

All sequencing data are available in the NCBI Gene Expression Ominibus under SuperSeries GSE243158. The secure token for reviewer access is wfolcuqovnwxnkd.

Human research participants

Policy information about studies involving human research participants and Sex and Gender in Research.

Reporting on sex and gender	Yes, studies are performed in samples that have a uterus and are of female sex.
Population characteristics	Samples are maintained using de-identified codes to preserve confidentiality. Endometrial samples were obtained from women with confirmed endometriosis (n= 7, mean age, 36.7 +/- 6.9) or from women without endometriosis (n= 7, mean age, 38.4 +/- 5.3) undergoing endometrial biopsies or hysterectomies. Samples categorized in the normal group were free of endometriosis, according to pathology examination reports.
Recruitment	All patients were enrolled prior to surgery by providing informed consent.
Ethics oversight	Study was approved by the Baylor College of Medicine Institutional Review Board, H-21138.

Note that full information on the approval of the study protocol must also be provided in the manuscript.

Field-specific reporting

Please select the one below that is the best fit for your research. If you are not sure, read the appropriate sections before making your selection.

☒ Life sciences ☐ Behavioural & social sciences ☐ Ecological, evolutionary & environmental sciences

For a reference copy of the document with all sections, see nature.com/documents/nr-reporting-summary-flat.pdf

Life sciences study design

All studies must disclose on these points even when the disclosure is negative.

Sample size	FThe sample size was determined based on a power analysis calculation that took into account various paraments, such as effect size, standard deviation, type 1 error and direction of effect. Sample size was chosen using a priori power tests and in consultation with bioinformatics specialist.
Data exclusions	Not applicable
Replication	All studies were perfomed using more than three biological replicates and with two to three technical replicates.
Randomization	N/A
Blinding	Because the analysis was being performed on patient samples according to disease subtype, and the differences in gene expression and protein expression were based on those differences, blinding was not performed.

Reporting for specific materials, systems and methods

We require information from authors about some types of materials, experimental systems and methods used in many studies. Here, indicate whether each material, system or method listed is relevant to your study. If you are not sure if a list item applies to your research, read the appropriate section before selecting a response.

Materials & experimental systems

n/a	Involved in the study
☐	☒ Antibodies
☐	☒ Eukaryotic cell lines
☒	☐ Palaeontology and archaeology
☒	☐ Animals and other organisms
☒	☐ Clinical data
☒	☐ Dual use research of concern

Methods

n/a	Involved in the study
☐	☒ ChIP-seq
☒	☐ Flow cytometry
☒	☐ MRI-based neuroimaging

Antibodies

Antibodies used

pSMAD1/5, Cell Signaling, 9516; SMAD1, Invitrogen, 38-5400; SMAD5, Proteintech 12167-1-AP; GAPDH, Proteintech HRP-60004; Vimentin, Cell Signaling 5741; SMAD4, Abcam ab40759; H3K27Ac, Cell Signaling 8173; KRT-8, DSHB TROMA-I; FOXJ1, Sigma HPA005714

Validation

1) pSMAD1/5, Cell Signaling #9516, IHC 1:200; this product is validated by the manufacturer and has 376 citations.

2) SMAD1, Invitrogen, 38-5400; WB 1:1000; is validated by the manufacturer, is shown to be highly specific, and has 20 associated references

2) SMAD5, Proteintech 12167-1-AP, WB 1:1000; is validated by the manufacturer for western blot and other applications and has 18 published references

3) GAPDH, Proteintech HRP-60004, WB 1:5000; is validated by the manufacturer and has 518 references.

4) Vimentin, Cell Signaling 5741, IF 1:200; This antibody is validated for various applications by the manufacturer and has 2562 citations in the literature.

5) SMAD4, Abcam #Ab40759, 0.678μg/reaction; SMAD4 antibody has been used for various applications and validated by the manufacturer. It has 136 citations.

6) H3K27Ac, Cell Signaling 8173, CUT&RUN 1:50; is validated by the manufacturer for various applications and has 379 associated citations.

7) CK8, DSHB TROMA-I, IF 1:50; is validated for immunostaining by the manufacturer and has been widely used in the literature for many years.

8) FOXJ1, Sigma HPA005714, IHC, IF 1:100; is validated for IHC and Western Blot by the manufacturer and has 20 published citations

Eukaryotic cell lines

Policy information about cell lines and Sex and Gender in Research

Cell line source(s)

Only primary, patient-derived cells were used for our studies. All studies were performed in cells with less than 6 passages.

Authentication

Describe the authentication procedures for each cell line used OR declare that none of the cell lines used were authenticated.

Mycoplasma contamination

Cells were routinely tested and cultured in the presence of Primocin to avoid mycoplasma.

Commonly misidentified lines
(See ICLAC register)

Name any commonly misidentified cell lines used in the study and provide a rationale for their use.

ChIP-seq

Data deposition

☒ Confirm that both raw and final processed data have been deposited in a public database such as GEO.

☒ Confirm that you have deposited or provided access to graph files (e.g. BED files) for the called peaks.

Data access links
May remain private before publication.

All sequencing data are available in the NCBI Gene Expression Ominibus under SuperSeries GSE243158

Files in database submission

GSM7777795 Normal endometrial stroma, Replicate 2, Day0
GSM7777796 Normal endometrial stroma, Replicate 2, Day2
GSM7777797 Normal endometrial stroma, Replicate 2, Day4
GSM7777798 Normal endometrial stroma, Replicate 2, Day6
GSM7777799 Normal endometrial stroma, Replicate 2, Day8
GSM7777800 Normal endometrial stroma, Replicate 4, Day0
GSM7777801 Normal endometrial stroma, Replicate 4, Day2
GSM7777802 Normal endometrial stroma, Replicate 4, Day4
GSM7777803 Normal endometrial stroma, Replicate 4, Day6
GSM7777804 Normal endometrial stroma, Replicate 4, Day8
GSM7777805 Normal endometrial stroma, Replicate 6, Day0
GSM7777806 Normal endometrial stroma, Replicate 6, Day2
GSM7777807 Normal endometrial stroma, Replicate 6, Day4
GSM7777808 Normal endometrial stroma, Replicate 6, Day6
GSM7777809 Normal endometrial stroma, Replicate 6, Day8
GSM7777810 Normal endometrial stroma, Replicate 7, Day0
GSM7777811 Normal endometrial stroma, Replicate 7, Day2
GSM7777812 Normal endometrial stroma, Replicate 7, Day4
GSM7777813 Normal endometrial stroma, Replicate 7, Day6
GSM7777814 Normal endometrial stroma, Replicate 7, Day8
GSM7777815 Endometriosis stroma, Replicate 1, Day0
GSM7777816 Endometriosis stroma, Replicate 1, Day2
GSM7777817 Endometriosis stroma, Replicate 1, Day4
GSM7777818 Endometriosis stroma, Replicate 1, Day6
GSM7777819 Endometriosis stroma, Replicate 1, Day8
GSM7777820 Endometriosis stroma, Replicate 10, Day0
GSM7777821 Endometriosis stroma, Replicate 10, Day2
GSM7777822 Endometriosis stroma, Replicate 10, Day4
GSM7777823 Endometriosis stroma, Replicate 10, Day6
GSM7777824 Endometriosis stroma, Replicate 10, Day8
GSM7777825 Endometriosis stroma, Replicate 12, Day0
GSM7777826 Endometriosis stroma, Replicate 12, Day2
GSM7777827 Endometriosis stroma, Replicate 12, Day4
GSM7777828 Endometriosis stroma, Replicate 12, Day6
GSM7777829 Endometriosis stroma, Replicate 12, Day8
GSM7777830 Endometriosis stroma, Replicate 13, Day0
GSM7777831 Endometriosis stroma, Replicate 13, Day2
GSM7777832 Endometriosis stroma, Replicate 13, Day4
GSM7777833 Endometriosis stroma, Replicate 13, Day6
GSM7777834 Endometriosis stroma, Replicate 13, Day8
GSM7780019 Normal endometrium stroma, EPC, SMAD4, replicate 1
GSM7780020 Normal endometrium stroma, EPC, SMAD4, replicate 2
GSM7780021 Normal endometrium stroma, EPC, H3K27ac, replicate 1
GSM7780022 Normal endometrium stroma, EPC, H3K27ac, replicate 2
GSM7780023 Normal endometrium stroma, EPC, IgG, replicate 1
GSM7780024 Normal endometrium stroma, EPC, IgG, replicate 2
GSM7780025 Endometriosis endometrium stroma, EPC, SMAD4, replicate 1
GSM7780026 Endometriosis endometrium stroma, EPC, SMAD4, replicate 2
GSM7780027 Endometriosis endometrium stroma, EPC, H3K27ac, replicate 1
GSM7780028 Endometriosis endometrium stroma, EPC, H3K27ac, replicate 2
GSM7780029 Endometriosis endometrium stroma, EPC, IgG, replicate 1
GSM7780030 Endometriosis endometrium stroma, EPC, IgG, replicate 2
GSM7780031 Normal endometrial stroma with the treatment of scrambled siRNA and vehicle, replicate 1
GSM7780032 Normal endometrial stroma with the treatment of scrambled siRNA and EPC, replicate 1
GSM7780033 Normal endometrial stroma with the treatment of SMAD1/5 siRNA and EPC, replicate 1
GSM7780034 Normal endometrial stroma with the treatment of SMAD1/5 siRNA and vehicle, replicate 1
GSM7780035 Normal endometrial stroma with the treatment of scrambled siRNA and vehicle, replicate 4
GSM7780036 Normal endometrial stroma with the treatment of scrambled siRNA and EPC, replicate 4
GSM7780037 Normal endometrial stroma with the treatment of SMAD1/5 siRNA and EPC, replicate 4
GSM7780038 Normal endometrial stroma with the treatment of SMAD1/5 siRNA and vehicle, replicate 4
GSM7780039 Normal endometrial stroma with the treatment of scrambled siRNA and vehicle, replicate 5
GSM7780040 Normal endometrial stroma with the treatment of scrambled siRNA and EPC, replicate 5
GSM7780041 Normal endometrial stroma with the treatment of SMAD1/5 siRNA and EPC, replicate 5
GSM7780042 Normal endometrial stroma with the treatment of SMAD1/5 siRNA and vehicle, replicate 5

Genome browser session
(e.g. UCSC)

All sequencing data are available in the NCBI Gene Expression Ominibus under SuperSeries GSE243158

Methodology

Replicates

2 technical replicates were perfomed for each experiment.

Sequencing depth	Approximately 10 million reads were obtained per sequencing run.
Antibodies	SMAD4, Abcam ab40759; H3K27Ac, Cell Signaling 8173
Peak calling parameters	CUT&RUN peaks were called by Model-based Analysis of ChIP-Seq (MACS/2.0.10)
Data quality	Data was ensured to meet ENCODE data quality standards.
Software	Model-based Analysis of ChIP-Seq (MACS/2.0.10); ChIPseeker; DiffBind; BETA packages were used to analyze datasets.
